# Traditional Chinese Medicine Treatment for Androgenetic Alopecia Based on Animal Experiments: A Systematic Review and Meta-Analysis

**DOI:** 10.1155/2022/2588608

**Published:** 2022-10-11

**Authors:** Yanbo Liang, Jing Yuan, Nururshopa Eskander Shazada, Jun Jiang, Jianlin Wu

**Affiliations:** ^1^Shandong University of Traditional Chinese Medicine, Jinan 250355, Shandong, China; ^2^Beijing University of Chinese Medicine, Beijing 100029, China; ^3^University of South Bohemia, Ceske Budejovice, Vodnany, Ceske Budejovic 38925, Czech Republic

## Abstract

**Background:**

In the present study, we systematically evaluated the effects of Traditional Chinese Medicine (TCM) on androgenetic alopecia (AGA) in rodent models (rats and mice) to provide potential evidence for the treatment of AGA by TCM.

**Methods:**

Previous research papers focusing on the treatment of AGA by TCM were retrieved from various electronic databases (PubMed, Embase, The Cochrane Library, CNKI, Vip, Wanfang data, and CBM) up to October 30, 2021. Screening of the literature was performed independently, and data were extracted and assessed. A meta-analysis was performed using RevMan 5.3 software.

**Results:**

When compared with the model groups, a group of C57BL/6 mice treated with TCM showed an increase in the total number of hair follicles (mean difference [MD] = 11.99, 95% confidence interval [CI] [5.94,18.03], *P*=0.0001), as well as a decrease in serum testosterone (T) level (MD = −1.10, 95% CI [−1.43, −0.78], *P* < 0.00001), skin discoloration time (MD = −2.93, 95% CI [−4.03, −1.84], *P* < 0.00001), and skin hair growth time (MD = −3.16, 95% CI [−4.16, −2.16], *P* < 0.00001). Terminal hair/vellus hair also increased in TCM-treated AGA animals (MD = 3.02, 95% CI [2.05, 3.98], *P* < 0.00001). No significant difference was found in serum estradiol (E_2_) level, skin tissue E_2_ level, or skin tissue T level between the TCM-treated group and the model group.

**Conclusion:**

TCM can increase the total number of hair follicles and terminal hair/vellus hair ratio, and reduce skin discoloration time and skin hair growth time in AGA animal models. These effects may be related to the reduction of the serum T level in AGA animals. These conclusions need to be verified by high-quality studies as the current analysis may be affected by the number and quality of the studies identified.

## 1. Introduction

Androgenic alopecia (AGA) is a common form of hair loss both in men and women and is likely due to an excessive response to androgens. AGA is characterized by the gradual miniaturization of hair follicles, shortening of the hair growth period, and reduced number of hairs [1]. Clinical manifestations of AGA include smooth skin in the alopecia area, increased skin sebum, dandruff, and pruritus. Hair in the frontotemporal region and on top of the head becomes increasingly sparse or even fully detached with time [2]. AGA is the most common type of hair loss in clinical practice and accounts for 84.8% of all hair loss [3]. The condition can impact the usual interpersonal communications and social relations of those affected.

AGA can be treated by medication, hair transplantation, low-energy laser therapy, mesoderm therapy, autologous platelet-rich plasma injection therapy, and TCM treatment. Drug therapy is an important treatment for AGA patients, with both minoxidil and finasteride approved by the Food and Drug Administration (FDA) for treatment of alopecia [4–6]. However, the clinical efficacy of these drugs is limited and they are also associated with certain side effects. Thus, it is essential to develop safer and more efficient drugs to manage this condition.

Seborrheic alopecia has been studied in TCM for thousands of years, and was first recorded in the “Yellow Emperor's Inner Classic” work. Based on the diagnosis and treatment characteristics of the condition as a whole, as well as its different syndromes, TCM has achieved satisfactory curative effects in the treatment of AGA. Recently, researchers working on clinical trials in TCM have shown that TCM offers advantages in treating the AGA; however, the mechanism of the effect is not yet clear. Therefore, we performed a meta-analysis to evaluate treatment effects of TCM in AGA in order to investigate possible treatments. Evaluation of animal models is necessary to improve the quality of *in vivo* experiments as it connects basic research and clinical trials [7].

## 2. Materials and Methods

We followed the Preferred Reporting Items for Systematic Reviews and Meta-analyses (PRISMA) guidelines [8] (Supplementary Table 1) for this systematic review and meta-analysis.

### 2.1. Inclusion and Exclusion Criteria

#### 2.1.1. Types of Study

We collected previously published research reports from various electronic databases up to October 30, 2021, regardless of language. We also manually reviewed the bibliographies of narrative review articles which could not be retrieved by electronic searches. Conferences proceedings, dissertation abstracts, and other unpublished data were also considered in the analyses to ensure that no potential studies were overlooked.

#### 2.1.2. Types of Subject

Rodents (mice and rats) were selected as our research objects irrespective of their sex or strain. The rodents were developed as models for alopecia or seborrheic alopecia, with no limits placed on the methods by which the rodent models were developed.

#### 2.1.3. Types of Intervention

The models developed for alopecia or seborrheic alopecia were all treated with TCM. Drug dosage and modes of drug administration were excluded from the analysis. Untreated model groups were used as controls, and experiments were not limited to a specific time period.

#### 2.1.4. Types of Outcome

Outcomes analyzed were total number of hair follicles, serum estradiol (E_2_) level, serum testosterone (T) level, skin tissue E_2_ level, skin tissue T level, skin discoloration time, skin hair growth time, and terminal hair/vellus hair.

#### 2.1.5. Exclusion Criteria

The following exclusion parameters were set (i) reprinted literature; (ii) original and unpublished data that could not be obtained and extracted after contacting the authors; (iii) *in vitro* studies; (iv) intervention measures including other nontraditional Chinese medicine treatments such as acupuncture.

### 2.2. Search Strategy

We retrieved previous research papers from different electronic databases including the CNKI database, Wanfang database, Vip database, CBM, PubMed, Embase, and the Cochrane Library up to October 30, 2021 in both English and Chinese, irrespective of publication status. The collected research reports focused on the use of TCM in rodent models for the treatment of seborrheic alopecia. Chinese search terms included zhongyi, zhongyiyao, xiongjisuxingtuofa, zhiyixingtuofa, and fazhutuofa and English search terms included Traditional Chinese Medicine, rodents, androgenic alopecia, and seborrheic alopecia.

### 2.3. Literature Screening and Data Extraction

Two independent investigators screened, extracted, and cross-checked the data across the entire study period. In case of disagreement, a third party was consulted to arbitrate. The following data were extracted using a pre-established data extraction table i) basic study information (first author, title, publication year, animal species, body weight, sample size, modeling method, and composition of TCM); (ii) specific details of the intervention measures, including the dosage and course of treatment; (iii) relevant information on the assessment of risk of bias risk; and (iv) outcome indicators and outcome measurement data.

### 2.4. Methodology Quality Evaluation

Ten items recommended by the SYRCLE and animal experiments bias risk assessment tools were used for evaluation, including generation and/or application of distributed sequences; whether each baseline was identical; allocation concealment; whether animals were randomly allocated during the experiment; whether researchers were blinded; whether outcome evaluation was randomly selected; and whether a method of blinding was adopted for the results evaluator. It was also noted whether incomplete data were reported and whether study reporting was irrelevant to selective outcome reporting, as well as any other biases. The evaluation results were denoted as “yes”, “no,” and “uncertain”, representing low risk of bias, high risk of bias, and uncertain risk of bias, respectively.

### 2.5. Statistical Analysis

RevMan 5.3 software was used to perform the meta-analysis. The relative risk was calculated as an effective indicator to count the data, and the mean difference (MD) was used as an effective indicator for data measurement. Point estimates and 95% confidence intervals (CIs) of each effect quantity were given. Heterogeneity among included studies was analyzed using the *χ*^2^ test, and the magnitude of heterogeneity was judged quantitatively using I^2^. If no obvious heterogeneity was found among the results of each study (I^2^ ≤ 50%), a fixed-effect model was used for the meta-analysis. If there was significant heterogeneity obtained among the studies (I^2^ > 50%), the source of heterogeneity was further analyzed. After excluding obvious methodological heterogeneity, a random-effects model was used for meta-analysis, and a descriptive analysis was performed.

## 3. Results

### 3.1. Literature Screening Process and Results

**Figure 1:**
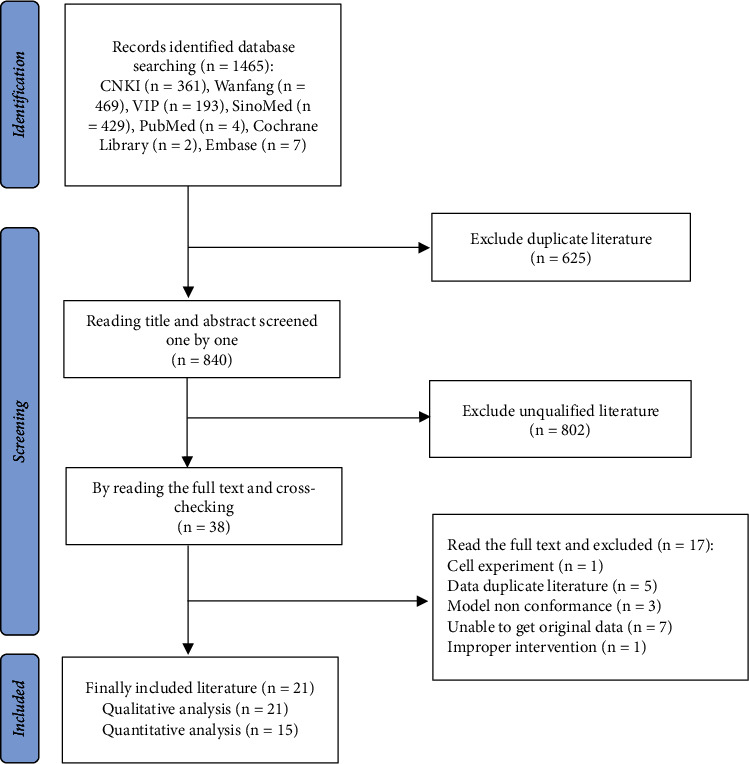
Flow diagram for identification and selection of included studies.

The results of the literature screening are shown in Figure 1. Twenty-one literature searches were included in the qualitative research, and 15 searches were included in the quantitative analysis.

### 3.2. Basic Characteristics of Included Studies

This analysis included 15 studies for quantitative analysis, all of which were taken from the Chinese literature. Of these, five studies used C57BL/6 mice [9–13], three used Wistar rats [14–16], two used Kunming mice [17, 18], two used Sprague–Dawley (SD) rats [19, 20], two used B6CBAF1/J mice [21, 22], and one study used white mice [23]. In terms of methods of model development, two studies used a subcutaneous injection of dihydrotestosterone solution [9, 14], eleven studies used a subcutaneous injection of testosterone propionate solution [10, 11, 15–23], and two studies used a topical application of testosterone propionate solution [12, 13]. The developed rodent models were included in both the AGA model and seborrheic alopecia model. Intervention methods involved external application and gavage. Outcome indicators mainly included total number of hair follicles, serum E_2_ level, serum T level, skin tissue E_2_ level, skin tissue T level, skin discoloration time, skin hair growth time, and terminal hair/vellus hair (Table 1).

### 3.3. Evaluation of Methodological Quality

Only three out of 15 studies used a random number table, and it was not possible to determine whether animals were randomly assigned during the rest of the experimental period. The selected studies did not mention allocation concealment or blinded methods. All collected research data reports were complete and unrelated to the results of selective reports (Table 2). Other sources of bias related to this study could not be determined.

① Whether the generation or application of allocation sequence was adequate; ② Whether each baseline was identical; ③ Whether the allocation concealment was sufficient; ④ Whether the animals were randomly allocated during the experiment; ⑤ Whether the researchers were blinded; ⑥ Whether the outcome evaluation was randomly selected; ⑦ Whether the result assessors were blinded; ⑧ Whether incomplete data were reported; ⑨ Whether the research report was irrelevant to the selective resulting report; ⑩ Whether there was no other bias.

### 3.4. Meta-Analysis Results

#### 3.4.1. Total Number of Follicles

**Figure 2:**
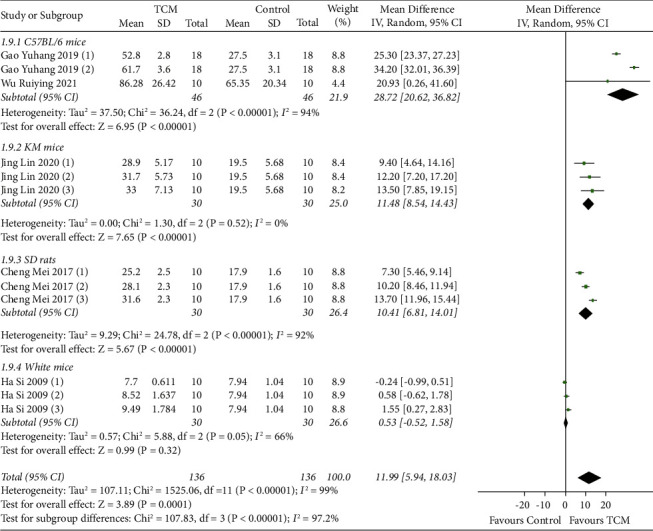
Meta-analysis results of total number of hair follicles for TCM group vs. model group.

Five studies reported the total number of hair follicles, with subgroup analyses based on animal species [9, 12, 17, 19, 23]. The results of a random-effect model meta-analysis showed that the total number of hair follicles increased in the TCM group in C57BL/6 mice (MD = 28.72, 95% CI [20.62, 36.82], *P* < 0.00001), Kunming mice (MD = 11.48, 95% CI [8.54, 14.43], *P* < 0.00001), and SD rats (MD = 10.41, 95% CI [6.81, 14.01], *P* < 0.00001) compared with the model group. No significant difference was found in the total number of hair follicles between the TCM group and the model group (MD = 0.53, 95% CI [−0.52, 1.58], *P*=0.32) (Figure 2).

#### 3.4.2. Serum E_2_ Level

**Figure 3:**
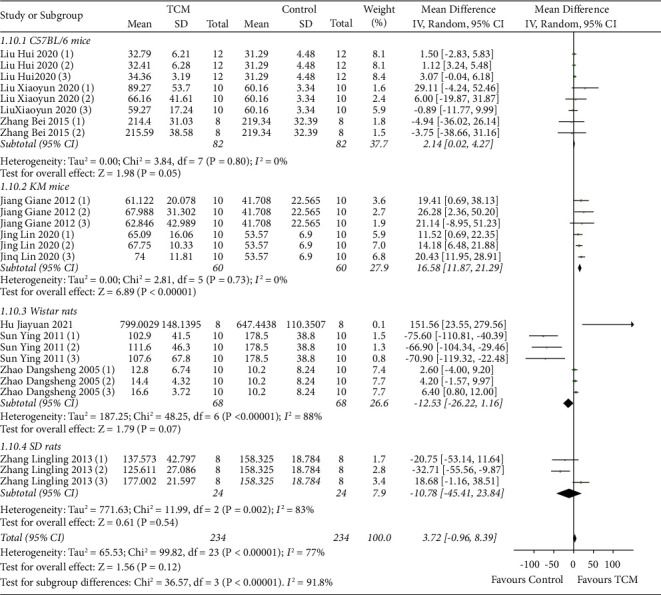
Meta-analysis of TCM group vs. model group on serum E2 levels.

Nine studies reported serum E_2_ levels with subgroup analyses based on animal species [10, 11, 13–18, 20]. The results of the random-effects model meta-analysis demonstrated that the level of serum E2 increased in the TCM group in Kunming mice (MD = 16.58, 95% CI [11.87, 21.29], *P* < 0.00001) compared with the model group. No significant differences in serum E_2_ levels were found in the TCM group compared with the model group in C57BL/6 mice (MD = 2.14, 95% CI [0.02, 4.27], *P* < 0.00001), Wistar rats (MD = −12.53, 95% CI [−26.22, 1.16], *P* < 0.00001), or SD rats (MD = −10.78, 95% CI [−45.41, 23.84], *P* < 0.00001) (Figure 3).

#### 3.4.3. Serum T Levels

**Figure 4:**
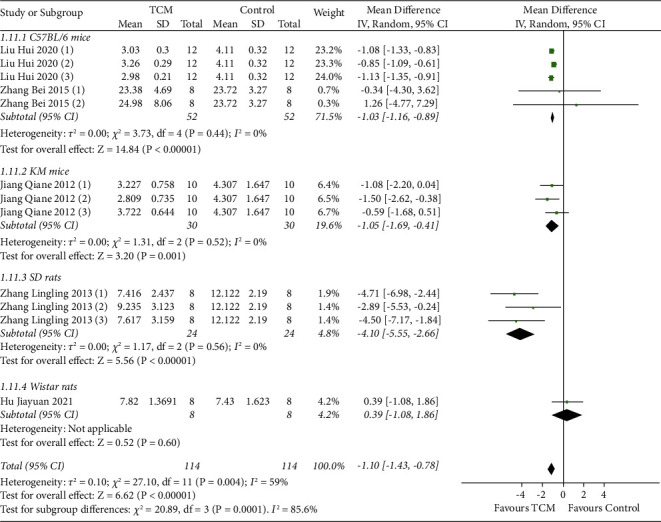
Meta-analysis results of TCM group vs. model group on serum T levels.

Five studies reported serum T levels with subgroup analyses performed according to animal species [11, 13, 14, 18, 20]. The results of the random-effects model meta-analysis showed that TCM reduced serum T levels in C57BL/6 mice (MD = −1.03, 95% CI [−1.16, −0.89], *P* < 0.00001), Kunming mice (MD = -1.05, 95% CI [-1.69, -0.41], *P*=0.001), and SD rats (MD = −4.10, 95% CI [−5.55, −2.66], *P* < 0.00001) compared with the model group. There was no statistically significant difference in serum T levels between the TCM group and the model group (MD = 0.39, 95% CI [−1.08, 1.86], *P*=0.60) (Figure 4).

#### 3.4.4. Skin Tissue E_2_ Levels

**Figure 5:**
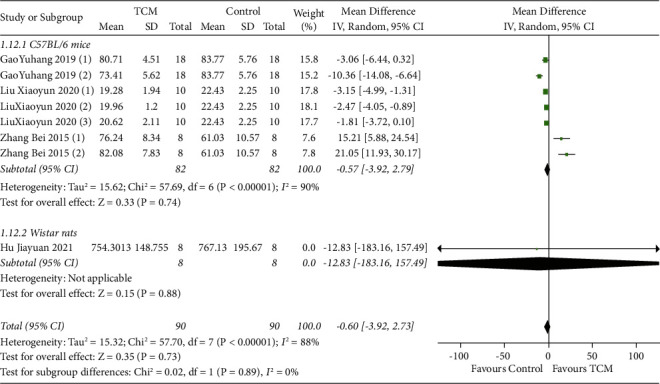
Meta-analysis results of TCM group vs. model group on E2 levels in skin tissue.

Four studies reported skin tissue E_2_ levels with subgroup analyses based on animal species [10, 12–14]. The results of the random-effects model meta-analysis showed that TCM had no statistically significant effect on skin tissue E_2_ levels in C57BL/6 mice (MD = −0.57, 95% CI [−3.92, 2.79], *P*=0.74) or Wistar rats (MD = −12.83, 95% CI [−183.16, 157.49], *P*=0.88) compared with the model group (Figure 5).

#### 3.4.5. Skin Tissue T Levels

**Figure 6:**
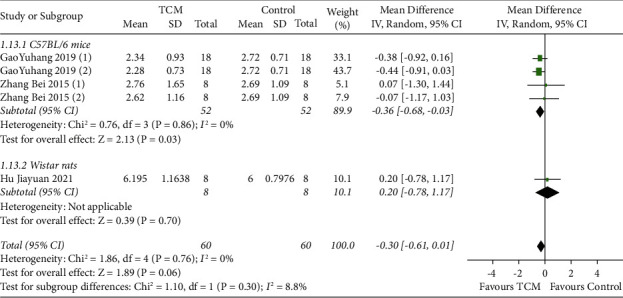
Meta-analysis result of TCM group vs. model group on T levels in skin tissue.

Three studies reported skin tissue T levels with subgroup analyses based on animal species [12–14]. The results of fixed-effect model meta-analysis showed that TCM reduced skin tissue T levels in C57BL/6 mice (MD = −0.36, 95% CI [−0.68, −0.03], *P*=0.03) compared with the model group. No statistically significant difference between the TCM and model groups was found in skin tissue T levels in Wistar rats (MD = 0.20, 95% CI [−0.78, 1.17], *P*=0.70) (Figure 6).

#### 3.4.6. Skin Discoloration Time

**Figure 7:**
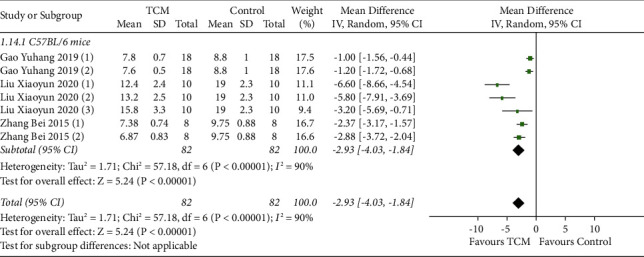
Meta-analysis result of TCM group vs. model group on skin discoloration time.

Three studies reported skin discoloration time with subgroup analyses based on animal species [10, 12, 13]. The results of the random-effect model meta-analysis showed that TCM reduced skin discoloration time in C57BL/6 mice (MD = −2.93, 95% CI [−4.03, −1.84], *P* < 0.00001) compared with the model group (Figure 7).

#### 3.4.7. Skin Hair Growth Time

**Figure 8:**
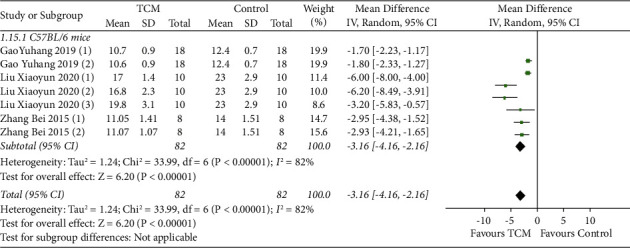
Meta-analysis result of TCM group vs model group on skin hair growth time.

A total of three studies reported skin hair growth time with subgroup analyses based on animal species [10, 12, 13]. The results of the random-effect model meta-analysis showed that TCM reduced skin hair growth time in C57BL/6 mice (MD = −3.16, 95% CI [−4.16, −2.16], *P* < 0.00001) compared with the model group (Figure 8).

#### 3.4.8. Terminal Hair/Vellus Hair

**Figure 9:**
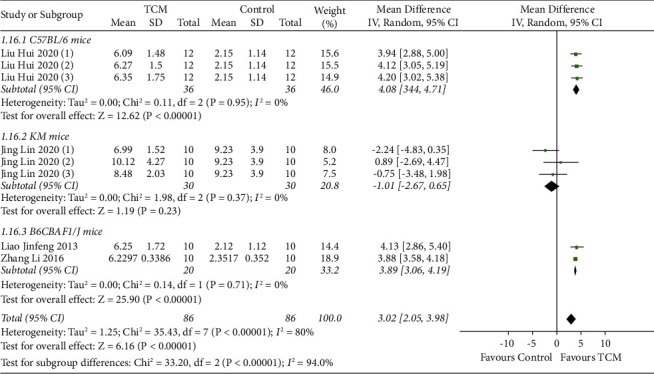
Meta-analysis result of TCM group vs. model group on terminal hair/vellus hair.

A total of four studies reported terminal hair/vellus hair with subgroup analyses based on animal species [11, 17, 21, 22]. The results of the random-effects model showed that TCM increased the terminal hair/vellus hair ratio in C57BL/6 mice (MD = 4.08, 95% CI [3.44, 4.71], *P* < 0.00001) and B6CBAF1/J mice (MD = 3.89, 95% CI [3.60, 4.19], *P* < 0.00001) compared with the model group. There was no statistically significant difference between the TCM group and model group in terminal hair/vellus hair in Kunming mice (MD = −1.01, 95% CI [−2.67, 0.65], *P*=0.23) (Figure 9).

### 3.5. Publication Bias

**Figure 10:**
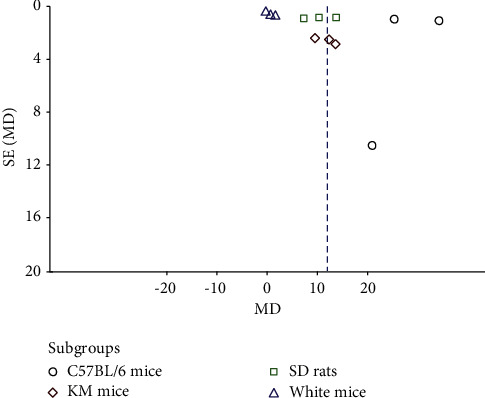
Funnel plot of analysis of the number of hair follicles.

A funnel plot was drawn based on the outcome index of the total number of hair follicles in the TCM group versus the model group (Figure 10). The results demonstrated that the distribution of each study on both sides of the funnel plot was asymmetric, thus indicating the existence of publication bias.

## 4. Discussion

To the best of our knowledge, this article is the first to use a meta-analysis to conduct a systematic review of the effects of TCM in animal models of AGA. Previous meta-analyses by You [24] and Wang [25] evaluated the curative efficacy and safety of TCM for treating AGA in clinical patients. We chose to evaluate research from animal experiments as it is very important to interrogate animal data thoroughly in order to improve the quality of animal research and guide clinical research. Our conclusions are consistent with previous clinical meta-analyses showing that TCM could be an effective and safe complementary therapy for AGA treatment.

### 4.1. Principal Findings

The outcomes of our study show that TCM can effectively increase the total number of hair follicles and the ratio of terminal hair to vellus hair, as well as decrease skin discoloration time and skin hair growth time. At the same time, TCM can also reduce serum T levels in animals with AGA. A reversal of the gradual miniaturization of hair follicles has consistently been shown to increase terminal hair density, thereby improving hair loss [5, 7]. However, owing to the publication bias in the outcome index, additional large-scale and high-quality trials are required for further verification and to obtain more reliable evidence.

### 4.2. TCM Understanding of Hair Loss

Chinese classic herbal formulas documented in the ancient Chinese medical literature have been widely used in AGA for centuries [24]. Examples include the “Yellow Emperor's Inner Classic” during the Warring States Period [26], “Treatise on Febrile Diseases” during the three Kingdoms period, and “Compendium of Materia Medica” during the Ming Dynasty [27]. For example, the “Yellow Emperor's Inner Classic” states that “the hair is outside the kidney essence, and when the essence and blood are sufficient, the hair is thick and shiny.” This clearly suggests that the growth of hair is related to the rise and fall of the essence and blood in the kidney and the filling of qi and blood.

Modern TCM has summarized and developed previous knowledge of seborrheic alopecia, and believes that this disease is closely related to the spleen, lung, kidney, liver, phlegm stagnation, and dampness-heat [28–33]. Most practitioners divide seborrheic alopecia into four syndrome types as follows (i) damp and heat fumigation; (ii) blood heat and wind dryness; (iii) blood deficiency and wind dryness; and (iv) liver and kidney deficiency. The corresponding treatment methods can be summarized as invigorating the spleen, clearing dampness, and clearing heat; cooling blood and moistening dryness; nourishing blood and removing wind and moistening dryness; and nourishing the liver and kidney. A number of researchers have looked at the causes and treatments of AGA within the context of TCM. For example, Liu suggests that the etiology of AGA is responsible for pathogenic dampness, while basic pathogenesis is pathogenic dampness obstructing sweat pores [34]. Jia believes that AGA is caused by deficiency of the spleen and stomach, up-flooding of damp-turbidity, lung-wei insecurity, and disharmony of blood based on the theory of “spleen and stomach deficiency with lung disease” [35]. Xuan formed the academic process of “regulating yin and yang” in the diagnosis and treatment of skin diseases through clinical practice and scientific research exploration [36]. Yang showed that seborrheic alopecia is caused by heat from the stomach and intestine dampness fumigating upward to the head and face, thereby invading hair roots and causing the hair to fall out [37]. Treatment is based on heat clearing methods and removal of dampness, strengthening the spleen and expelling wind, supplemented by cooling blood and promoting blood circulation. According to the investigations of Wang, seborrheic alopecia is caused by problems in the spleen and kidney, suggesting that the spleen should first be strengthened and warmed to eliminate dampness, resolving phlegm, eliminating lipids, and increasing blood flow [38]. Finally, the studies of Cheng presume that dampness-heat constitution is the main cause for alopecia seborrheic, while stasis-turbidity causing decayed hair is the main pathogenesis [33].

### 4.3. Modern Medicine Understanding of Hair Loss

AGA is characterized by progressive hair follicular miniaturization [39], caused by the action of androgens on the epithelial cells of genetically susceptible hair follicles in androgen-dependent areas [40]. The overall goal of AGA treatment is to arrest miniaturization and improve hair density. Systemic and local conversion of testosterone to dihydrotestosterone (DHT) by 5-*α*-reductase (5AR) enzyme is the main factor responsible for the underlying pathological mechanism in AGA [41, 42]. Therapeutic targets reduce DHT production, cause vasodilatory effects, trigger anagen, prolong anagen, and subdue inflammation [43]. Other factors that affect seborrheic alopecia include genetics, endocrine, mental stress, overuse of the brain, staying up too late, and improper diet, for example. However, the effects of androgen and 5*α*-reductase on hair follicles have always been considered the most important factors in AGA [11, 44].

Minoxidil, finasteride, and low-level laser light therapy (LLLT) are FDA-approved therapy options for AGA, with a significant effect [45–52]. In addition, dutasteride [41, 53], platelet-rich plasma (PRP) [54–56], photobiomodulation [50], and microneedling [57] are further treatment options for AGA. However, these treatment options are cost-intensive and also require lifelong treatment, thus may have side effects. The available literature suggests a role of herbal drugs in inhibiting 5‐alpha‐reductase enzyme and therefore reducing hair loss. This can be further potentiated since herbal drugs exhibit fewer side effects [58].

Regarding the signaling pathway of TCM for hair loss, a study by Dou [59] demonstrated that monomeric components in Chinese medicine can act through various pathways such as Wnt, MAPKs, PI3K/Akt, androgen receptors, Fas/FasL to initiate or terminate a program of related factors. These factors can competitively inhibit or synergistically activate related receptors and link proliferation or apoptosis of the hair follicle cells or dermal papilla cells. Therefore, a number of hairs remain in the anagen phase and achieve the effect of hair loss treatment and hair loss prevention.

### 4.4. Limitations

Our present study has a number of limitations. (i) All studies included in this analysis were sourced from the Chinese literature. As no manual search was conducted, some of the literature may have been overlooked, and the research conclusions may therefore be subject to publication bias. Indeed, a funnel plot of the total number of the hair follicles showed publication bias. (ii) The included literature did not always mention allocation concealment and blinding methods, potentially presenting selection and implementation bias. (iii) There was no consistency in the types of model animals included in this study, as well as inconsistency in the treatment cycles. Also, the interventions measured in the model groups were not similar, potentially leading to high heterogeneity and low research quality. (iv) Lack of reports of morbidity and mortality rates and adverse reactions of experimental animals were another significant drawback to this meta-analysis. Taking all the limitations into account, the conclusions of the present study require further corroboration.

### 4.5. Implications for Future Trials

More scientific, rigorous, and reasonable high-quality animal experiments should be carried out in the future. The studies should include a standardized and scientific experimental scheme, describing in detail the methods of generating allocation sequences, as well as methods of concealing the random allocation and blinding. Further reports of adverse reactions and morbidity and mortality rates are also required. Selection of standardized markers with important effects on AGA should be undertaken so that the mechanism of action of TCM on AGA may be fully understood, allowing TCM to be applied in clinical practice.

## 5. Conclusions

Androgenic alopecia is a genetically predetermined disorder that is caused by an excessive response to androgens. The condition has attracted considerable attention in society as it has a substantial effect on the social confidence of individuals. The clinical efficacy of drugs approved by the FDA is limited, and these drugs lead to side effects. Although TCM has been used to address these issues for centuries, the mechanism of action of TCM remains unclear. Our present study is the first of its kind and demonstrates that TCM can be potentially used to treat the AGA, although further studies are essential to validate the results.

## Figures and Tables

**Table 1:** General characteristics of included studies.

Studies included	Experimental animals/strains	Molding methods	Animal models	Group (n)	Intervention measures	Course of treatment	Outcome indicators	Chinese medicine ingredients
Wu Ruiying [9]	Male C57BL/6 mice, (20±2) g	Subcutaneous injection of dihydrotestosterone solution	Androgenetic alopecia model	Negative control group (10)	External saline	24 d	①	Nigella seeds, peach kernels, pomegranate seeds
				Model Set (10)	External saline			
				Positive control group (10)	Topical 5% Minoxidil Tincture			
				Experimental group (10)	External use compound Si Yadan hair growth tincture 0.4 ml/piece/day			

Liu Xiaoyun [10]	Male C57BL/6 mice, 18 ∼ 25 g	Subcutaneous injection of testosterone propionate solution	Androgenetic alopecia model	Blank group (10)	75% ethanol solution for external use	35 d	①②③④⑤⑦⑧	Ligustrum lucidum alcohol extract
				Model set (10)	75% ethanol solution for external use			
				Minoxidil group (10)	5% minoxidil solution for external use			
				Ligustrum lucidum low-dose group (10)	1 mg/piece/d for external use			
				Ligustrum lucidum medium dose group (10)	2 mg/piece/d for external use			
				Ligustrum lucidum high-dose group (10)	4 mg/piece/d for external use			

Liu Hui [11]	C57BL/6J mice, (20±2) g	Subcutaneous injection of testosterone propionate solution	Androgenetic alopecia model	Normal group (12)	Distilled water gavage	10 w	②③⑧	Tempeh
				Model set (12)	Distilled water gavage			
				Positive control group (12)	Aqueous solution of finasteride tablets gavage			
				Tempeh low-dose group (12)	3.25 g/kg/d gavage			
				Tempeh medium dose group (12)	6.50 g/kg/d gavage			
				Tempeh high-dose group (12)	13.00 g/kg/d gavage			

Gao Yuhang [12]	Male C57BL/6J mice, 18∼20 g	Application of 0.05% testosterone solution	Seborrheic Alopecia Model	Normal group (18)	Normal saline for external use	17 d	①④⑤⑥⑦	Dried ginger, five fingers peach
				Model Group (18)	Normal saline for external use			
				Finasteride group (18)	2% finasteride topical			
				Dry ginger Wuzhifang low-dose group (18)	20 mg/mL concentration 0.1 mL external use			
				Dry Ginger Wuzhifang High-Dose Group (18)	50 mg/mL concentration 0.1 mL external use			

Zhang Bei [13]	Male C57BL/6 mice, 15∼20 g	Application of 100 *μ*L of 0.05% testosterone solution	Seborrheic Alopecia Model	Normal group (10)	75% ethanol solution for external use	30 d	①②③④⑤⑥⑦	Arborvitae
				Model Set (10)	75% ethanol solution for external use			
				Positive group (10)	2% finasteride solution for topical use			
				Platycladus orientalis low-dose group (10)	20 mg/mL concentration 2 mg/only/d for external use			
				Arborvitae high-dose group (10)	50 mg/mL concentration 5 mg/only/d for external use			

Hu Jiayuan [14]	Male Wistar rats (220±20) g	Subcutaneous injection of dihydrotestosterone solution	Androgenetic alopecia model	Blank control group (8)	Saline gavage	60 d	②③④⑤	Tanshinone
				Model group (8)	saline gavage			
				Finasteride group (8)	0.12 mg/kg/d gavage			
				Tanshinone group (8)	0.24 g/kg/d gavage			

Sun Ying [15]	Male Wistar rats, (180±10) g	Subcutaneous testosterone propionate injection	Seborrheic alopecia model	Blank group (10)	Saline gavage	8 w	②③	Polygonum multiflorum, Salvia, Alisma, fried Atractylodes, Poria, Chuanxiong
				Model Set (10)	Saline gavage			
				Positive control group (10)	Spironolactone suspension 9 mg/kg gavage			
				Shengfaling granule low-dose group (10)	5 g/kg gavage			
				Shengfaling granules medium dosage group (10)	10 g/kg gavage			
				Shengfaling granule high-dose group (10)	15 g/kg gavage			

Zhao Dangsheng [16]	Wistar rats, half male and half female, (200±20) g	Subcutaneous testosterone propionate injection	Seborrheic Alopecia model	Normal control group (10)	Saline gavage	60 d	②③	Salvia, Polygonum multiflorum, Hawthorn, Ginseng leaves, Rehmannia glutinosa, Rhubarb, Dandelion, Polygonatum, Angelica sinensis, Licorice
				Model set (10)	saline gavage			
				Positive control group (10)	Spironolactone suspension 9 mg/kg gavage			
				Zhituoling low-dose group (10)	5 g/kg gavage			
				Zhituoling middle dose group (10)	10 g/kg gavage			
				Zhituoling high-dose group (10)	15 g/kg gavage			

Jing lin [17]	Kunming mice, half male and half female, (20±2) g	Subcutaneous testosterone propionate injection	Androgenetic alopecia model	Blank group (10)	—	30 d	①②③⑧	Astragalus, Arborvitae, Safflower
				Model set (10)	—			
				Ethanol group (10)	55% ethanol 0.24 ml for external use			
				Minoxidil Liniment set (10)	Minoxidil liniment 0.24 mL/pc			
				Low-dose group of nourishing qi and promoting blood circulation (10)	50 mg/mL concentration, 0.24 mL/piece			
				Middle dose group of tonifying qi and promoting blood circulation (10)	100 mg/mL concentration, 0.24 mL/piece			
				High-dose group of nourishing qi and promoting blood circulation (10)	200 mg/mL concentration, 0.24 mL/piece			

Jiang Qian [18]	Kunming mice, half male and half female, 18∼22 g	Subcutaneous testosterone propionate injection	Androgenetic alopecia model	Blank control group (10)	—	60 d	②③	Dried Ginger, Chuanxiong, Safflower, Sichuan Pepper, Parsnip, Scutellaria, Angelica, Vitex
				Model set (10)	75% ethanol 0.2 mL smear			
				Positive control group (10)	101 hair tonic 0.2 mL smear			
				Chinese medicine hair growth liquid low-dose group (10)	0.1 mL/smear only			
				Medium dose group of traditional Chinese medicine hair growth liquid (10)	0.15 mL/smear only			
				High-dose group of traditional Chinese medicine hair growth liquid (10)	0.2 mL/apply only			

Cheng Mei [19]	Male SD rats, (200±20) g	Subcutaneous injection of testosterone propionate	Androgenetic alopecia model	Blank group (10)	10 mL distilled water to apply	56 d	①③	Alum, Saponin, Sophora flavescens, Treats, Neem root bark, Arborvitae leaves, Kochia, Mulberry bark, Ephedra root
				Model set (10)	10 mL distilled water to apply			
				Minoxidil Tincture set (10)	0.05 g/mL minoxidil tincture spread			
				Compound saponin lotion low-dose group (10)	0.25 g/mL concentration 10 mL smear			
				Compound saponin lotion medium dose group (10)	0.5 g/mL concentration 10 mL smear			
				Compound saponin lotion high-dose group (10)	2 g/mL concentration 10 mL smear			

Zhang Lingling [20]	SD rats, half male and half female, (200±20) g	Subcutaneous injection of testosterone propionate solution	Seborrheic Alopecia model	Normal control group (8)	Saline smear	60 d	②③	Ginseng, Astragalus, Dried Ginger, Chuanxiong, Angelica, Sichuan Pepper, Parsnip
				Degreasing model group (8)	Saline smear			
				101 Hair Tonic Group (8)	1 mL/smear only			
				Chinese medicine hair growth liquid low-dose group (8)	1 mL/smear only			
				Medium dose group of traditional Chinese medicine hair growth liquid (8)	2 mL/smear only			
				High-dose group of traditional Chinese medicine hair growth liquid (8)	4 mL/apply only			

Zhang Li [21]	Male B6CBAF1/J mice, about 20 g	Subcutaneous injection of testosterone propionate	Androgenetic alopecia model	Blank group (10)	—	9 w	③⑧	Usma grass
				Model set (10)	—			
				Normal saline group (10)	Normal saline for external use			
				Minoxidil group (10)	3% Minoxidil solution for external use			
				Usma grass group (10)	Usma grass fresh juice for external use			

Liao Jinfeng [22]	Male B6CBAF1/J mice, 20∼22 g	Subcutaneous testosterone propionate injection	Androgenetic alopecia model	Blank group (10)	—	4 w	②③⑧	Oleanolic acid
				Model set (10)	—			
				Matrix group (10)	External use of blank solution without oleanolic acid added			
				Positive control group (10)	5% concentration 0.2 mL minoxidil solution for external use			
				Oleanolic Acid (10)	1% concentration 0.2 mL oleanolic acid solution external use			

Haschmeg [23]	White mice, half male and half female, 22∼28 g	Subcutaneous testosterone propionate injection	Seborrheic Alopecia Model	Negative control group (10)	—	45 d	①	Tujingpi, Polygonum multiflorum, safflower, Shudi, Salvia
				Positive control group (10)	External use of hair growth tincture			
				Shufakang low-dose group (10)	0.3 mL for external use			
				Shufakang middle dose group (10)	0.45 mL for external use			
				Shufakang high-dose group (10)	0.6 mL for external use			

① Total number of hair follicles; ② Serum E2 level; ③ Serum T level; ④ Skin tissue E2 level; ⑤ Skin tissue T level; ⑥ Skin discoloration time; ⑦ Skin hair growth time; ⑧ Terminal hair/vellus hair.

**Table 2:** Bias risk assessment of included studies.

Included in the study	①	②	③	④	⑤	⑥	⑦	⑧	⑨	⑩
Wu Ruiying [9]	‘Random' only mentioned	Yes	Indeterminacy	Indeterminacy	Indeterminacy	Indeterminacy	Indeterminacy	Yes	Yes	Indeterminacy
Liu Xiaoyun [10]	‘Random' only mentioned	Yes	Indeterminacy	Indeterminacy	Indeterminacy	Indeterminacy	Indeterminacy	Yes	Yes	Indeterminacy
Liu Hui [11]	‘Random' only mentioned	Yes	Indeterminacy	Indeterminacy	Indeterminacy	Indeterminacy	Indeterminacy	Yes	Yes	Indeterminacy
Gao Yuhang [12]	‘Random' only mentioned	Yes	Indeterminacy	Indeterminacy	Indeterminacy	Indeterminacy	Indeterminacy	Yes	Yes	Indeterminacy
Zhang Bei [13]	‘Random' only mentioned	Yes	Indeterminacy	Indeterminacy	Indeterminacy	Indeterminacy	Indeterminacy	Yes	Yes	Indeterminacy
Hu Jiayuan [14]	Random digital table method	Yes	Indeterminacy	Indeterminacy	Indeterminacy	Indeterminacy	Indeterminacy	Yes	Yes	Indeterminacy
Sun Ying [15]	‘Random' only mentioned	Yes	Indeterminacy	Indeterminacy	Indeterminacy	Indeterminacy	Indeterminacy	Yes	Yes	Indeterminacy
Zhao Dangsheng [16]	‘Random' only mentioned	Yes	Indeterminacy	Indeterminacy	Indeterminacy	Indeterminacy	Indeterminacy	Yes	Yes	Indeterminacy
Jinglin [17]	‘Random' only mentioned	Yes	Indeterminacy	Indeterminacy	Indeterminacy	Indeterminacy	Indeterminacy	Yes	Yes	Indeterminacy
Jiang Qian [18]	‘Random' only mentioned	Yes	Indeterminacy	Indeterminacy	Indeterminacy	Indeterminacy	Indeterminacy	Yes	Yes	Indeterminacy
Cheng Mei [19]	Random digital table method	Yes	Indeterminacy	Indeterminacy	Indeterminacy	Indeterminacy	Indeterminacy	Yes	Yes	Indeterminacy
Zhang Lingling [20]	‘Random' only mentioned	Yes	Indeterminacy	Indeterminacy	Indeterminacy	Indeterminacy	Indeterminacy	Yes	Yes	Indeterminacy
Zhang Li [21]	‘Random' only mentioned	Yes	Indeterminacy	Indeterminacy	Indeterminacy	Indeterminacy	Indeterminacy	Yes	Yes	Indeterminacy
Liao Jinfeng [22]	Random digital table method	Yes	Indeterminacy	Indeterminacy	Indeterminacy	Indeterminacy	Indeterminacy	Yes	Yes	Indeterminacy
Hasgemeige [23]	‘Random' only mentioned	Yes	Indeterminacy	Indeterminacy	Indeterminacy	Indeterminacy	Indeterminacy	Yes	Yes	Indeterminacy

① Whether the generation or application of allocation sequence was adequate; ② Whether each baseline was identical; ③ Whether the allocation concealment was sufficient; ④ Whether the animals were randomly allocated during the experiment; ⑤ Whether the researchers were blinded; ⑥ Whether the outcome evaluation was randomly selected; ⑦ Whether the result assessors were blinded; ⑧ Whether incomplete data were reported; ⑨ Whether the research report was irrelevant to the selective resulting report; ⑩ Whether there was no other bias.

## Data Availability

The data used to support the findings of this study are available from the corresponding author upon request.

## References

[1] Premanand A., Reena Rajkumari B. (2018). Androgen modulation of Wnt-*β*catenin signaling in androgenetic alopecia. *Archives of Dermatological Research*.

[2] Gao J. Y., Liu H. J., Xie Y. B. (2017). Clinical efficacy observation of abdominal acupuncture combined with moxibustion in the treatment of male seborrheic alopecia with deficiency of qi and blood. *Modern Journal of Integrated Traditional Chinese and Western Medicine*.

[3] Jain R., De-Eknamkul W. (2014). Potential targets in the discovery of new hair growth promoters for androgenic alopecia. *Expert Opinion on Therapeutic Targets*.

[4] Suchonwanit P., Iamsumang W., Leerunyakul K. (2022). Topical finasteride for the treatment of male androgenetic alopecia and female pattern hair loss: a review of the current literature. *Journal of Dermatological Treatment*.

[5] Gupta A. K., Foley K. A. (2014). 5% Minoxidil: treatment for female pattern hair loss. *Skin Therapy Lett*.

[6] Wu W., Zhao H. W. (2021). Research progress of new drugs and drug therapy for androgenetic alopecia. *Journal of Tissue Engineering and Reconstructive Surgery*.

[7] Xu P., Lv Z. G. (2018). Systematic evaluation of experimental studies on the intervention of traditional Chinese medicine on autoimmune myasthenia gravis animals. *Journal of Traditional Chinese Medicine*.

[8] (2021). *PRISMA Transparent Reporting of Systematic Reviews and Meta-Analyses*.

[9] Wu R. Y. (2021). *Clinical and Experimental Study on the Treatment of Androgenic Alopecia with Compound Siyadan Tincture*.

[10] Liu X. Y., Wu X. S., Pan J. L. (2020). Effect of ethanol extract of Ligustri Lucidi Fructus on the hair growth of testosterone-induced alopecia mice. *Journal of Guangdong Pharmaceutical University*.

[11] Liu H., Dong W. T., Huo J. H. (2020). Studies on improvement effect of soybean meal on androgenetic alopecia in mice and its mechanism. *Chinese Journal of Traditionl Medical Science and Technology*.

[12] Gao Y. H., Lin X. M., Wu Y. N. (2019). Effect of Ganjiang Wuzhi decoction on hair growth in mice with seborrheic alopecia. *Traditional Chinese Drug Research & Clinical Pharmacology*.

[13] Zhang B. (2015). *The Screening of 5α-Reductase Inhibitor for Seborrheic Alopecia from Traditional Chinese Herbs and the Pharmacological Evaluation of Cacumen Platycladi*.

[14] Hu J. Y., Li N., Di J. (2021). Study on the therapeutic effect of Danshinone on androgenetic alopecia rat model. *China Journal of Leprosy and Skin Diseases*.

[15] Sun Y., Wu Z., Jing Y. (2011). The effect of Shengfaling on serum T, E2, T/E2 regulation and hair in experimental rats. *Jilin Journal of Traditional Chinese Medicine*.

[16] Zhao D. S., Li S. X., Zhao C. L. (2005). Regulation of “Zhituoling” on androgen in experimental rats and its effect on hair loss. *Journal of∙Gansu College of TCM*.

[17] Jing L., Yu Z. J., Hu T. T. (2020). Effects of buqi huoxue shengfa tincture on androgenetic alopecia in mice. *Chinese Traditional Patent Medicine*.

[18] Jiang J. E. (2012). *The Study of “Chinese Medicine Hair Restorer” Clinical and the Pharmacological Mechanism*.

[19] Cheng M., Yang C. Y., Yang W. X. (2017). Study on the therapeutical effect and mechanism of Compound Zaofan Lotion on androgenic alopecia. *Pharmacology and Clinics of Chinese Materia Medica*.

[20] Zhang L. L. (2013). *Pharmacodynamic Evaluation and Mechanism Research of Chinese Medicine Hair Restore on the Treatment of Seborrheic Alopecia*.

[21] Zhang L. (2016). *The Study of Isatis Indigotica Fort Treat Animal Model of Androgenetic Alopecia*.

[22] Liao J. F. (2013). *Oleanolic Acid on Androgen Alopecia Treatment Function of Mice*.

[23] Hasgemeige R. (2009). Experimental study on the treatment of seborrheic alopecia with the Chinese medicine Shufakang. *Journal of Northwest University for Nationalities (Natural Science)*.

[24] You Q., Li L., Ma X. (2019). Meta-analysis on the efficacy and safety of traditional Chinese medicine as adjuvant therapy for refractory androgenetic alopecia. *Evidence-based Complementary and Alternative Medicine*.

[25] Wang Q. H., Wang L. M., Qin Y. F. (2021). Systematic evaluation of traditional Chinese medicine in the treatment of seborrheic alopecia. *Zhejiang Journal of Traditional Chinese Medicine*.

[26] Zhao J., Zhang Y. M., Jing Y. L. (2014). Historical evolution on hair loss in treatment with traditional Chinese medicine. *World Journal of Integrated Traditional and Western Medicine*.

[27] Liu J. L., Sun L. Y. (2017). Clinical study of traditional Chinese medicine combined with minoxidil tincture in treatment of androgenic alopecia of liver and kidney deficiency syndrome. *Chinese Journal of Dermatovenereology of Integrated Traditional and Western Medicine*.

[28] Zhong R. B., Jia F. F., Wang H. H. (2018). Treatment of Seborrheic hair loss from liver spleen theory. *World Latest Medicine Information*.

[29] Pang Y. Y., Cao Y. (2018). Cao yi’s treatment of seborrheic alopecia from the lung. *Zhejiang Journal of Traditional Chinese Medicine*.

[30] Li S. J., Hang X. H., Lin X. R. (2020). Treating seborrheic alopecia from the liver. *Globel Traditional Chinese Medicine*.

[31] Lin X. Q., Ding W. Y., Jin T. (2018). Treatment of seborrheic alopecia from phlegm and blood stasis. *Zhejiang Journal of Traditional Chinese Medicine*.

[32] Jiang A., Yu M. (2018). Treatment of aeborrheic alopecia from deficiency, phlegm and blood stasis. *Journal of Sichuan of Traditional Chinese Medicine*.

[33] Xia J., Ni C. (2019). Treatment of aeborrheic alopecia from damp heat constitution. *Tianjin Journal of Traditional Chinese Medicine*.

[34] Shi N. N., Sun Z. Z., Lu M. C. (2021). Professor LIU Lanlin’s experience in treating seborrheic alopecia with the theory of syndrome differentiation of triple energizer. *Journal of Gansu University of Chinese Medicine*.

[35] Yang M. Y., Cao L. R., Jia Y. X. (2021). Experience introduction of JIA yuxin treating seborrheic alopecia based on ‘spleen and stomach deficiency with lung disease. *JOURNAL OF NEW CHINESE MEDICINE*.

[36] He Z. H., Lu C. J., Xuan G. W. (2021). TCM master XUAN Guo-wei’s experience in treating seborrheic alopecia with acupuncture and medicine based on the theory of blood stasis. *China Journal of Traditional Chinese Medicine and Pharmacy*.

[37] Zhao J., Yang Z. B., Li Z. S. (2020). Professor YANG zhibo’s experience in the treatment of seborrheic alopecia. *Journal of Traditional Chinese Medicine of hunan*.

[38] Wang Y. J., Wu J. D. (2020). Treating seborrheic alopecia from spleen. *Journal of Practical Traditional Chinese Internal Medicine*.

[39] Zhong S. X., Li C., Li Y., Fang S. J. (2021). Meta-analysis of platelet-rich plasma in the treatment of androgenetic alopecia. *Chinese Journal of Applied Mechanics*.

[40] Lolli F., Pallotti F., Rossi A. (2017). Androgenetic alopecia: a review. *Endocrine*.

[41] Arif T., Dorjay K., Adil M., Sami M. (2017). Dutasteride in androgenetic alopecia: an update. *Current Clinical Pharmacology*.

[42] Said M. A., Mehta A. (2018). The impact of 5*α*-reductase inhibitor use for male pattern hair loss on men’s health. *Current Urology Reports*.

[43] York K., Meah N., Bhoyrul B., Sinclair R. (2020). A review of the treatment of male pattern hair loss. *Expert Opinion on Pharmacotherapy*.

[44] Zhong S. X. (2020). *Turtle Plate Tincture Regulates Wnt/β-catenin Signal to Induce Hair Follicle Stem Cells to Prevent Androgenetic Alopecia*.

[45] Adil A., Godwin M. (2017). The effectiveness of treatments for androgenetic alopecia: a systematic review and meta-analysis. *Journal of the American Academy of Dermatology*.

[46] Lee S., Lee Y. B., Choe S. J. (2019). Adverse sexual effects of treatment with finasteride or dutasteride for male androgenetic alopecia: a systematic review and meta analysis. *Acta Dermato-Venereologica*.

[47] Zhou Z. B., Song S. Q., Gao Z. L., Wu J., Ma J., Cui Y. (2019). The efficacy and safety of dutasteride compared with finasteride in treating men with androgenetic alopecia: a systematic review and meta-analysis. *Clinical Interventions in Aging*.

[48] Gupta A. K., Charrette A. (2014). The efficacy and safety of 5*α*-reductase inhibitors in androgenetic alopecia: a network meta-analysis and benefit–risk assessment of finasteride and dutasteride. *Journal of Dermatological Treatment*.

[49] Chen L., Zhang J., Wang L., Wang H., Chen B. (2020). The efficacy and safety of finasteride combined with topical minoxidil for androgenetic alopecia: a systematic review and meta-analysis. *Aesthetic Plastic Surgery*.

[50] Gupta A. K., Carviel J. L. (2021). Meta-analysis of photobiomodulation for the treatment of androgenetic alopecia. *Journal of Dermatological Treatment*.

[51] Randolph M., Tosti A. (2021). Oral minoxidil treatment for hair loss: a review of efficacy and safety. *Journal of the American Academy of Dermatology*.

[52] Gupta A. K., Mays R. R., Dotzert M. S., Versteeg S. G., Shear N. H., Piguet V. (2018). Efficacy of non-surgical treatments for androgenetic alopecia: a systematic review and network meta-analysis. *Journal of the European Academy of Dermatology and Venereology*.

[53] Reguero-del Cura L., Durán-Vian C., de Quintana-Sancho A. (2020). RF-mesotherapy with dutasteride: a future alternative treatment for androgenetic alopecia. *Actas Dermo-Sifiliográficas*.

[54] Gupta A. K., Carviel J. L. (2017). Meta-analysis of efficacy of platelet-rich plasma therapy for androgenetic alopecia. *Journal of Dermatological Treatment*.

[55] Gupta A. K., Cole J., Deutsch D. P. (2019). Platelet-rich plasma as a treatment for androgenetic alopecia. *Dermatologic Surgery*.

[56] Giordano S., Romeo M., di Summa P., Salval A., Lankinen P. (2018). A meta-analysis on evidence of platelet-rich plasma for androgenetic alopecia. *International Journal of Trichology*.

[57] Gupta A. K., Quinlan E. M., Venkataraman M., Bamimore M. A. (2022). Microneedling for hair loss. *Journal of Cosmetic Dermatology*.

[58] Dhariwala M. Y., Ravikumar P. (2019). An overview of herbal alternatives in androgenetic alopecia. *Journal of Cosmetic Dermatology*.

[59] Dou J., Zhang Z., Xu X., Zhang X. (2022). Exploring the effects of Chinese herbal ingredients on the signaling pathway of alopecia and the screening of effective Chinese herbal compounds. *Journal of Ethnopharmacology*.

